# Bioenergetic Impairment in the Neuro-Glia-Vascular Unit: An Emerging Physiopathology during Aging

**DOI:** 10.14336/AD.2021.04017

**Published:** 2021-12-01

**Authors:** Minghao Yuan, Yangyang Wang, Shengyuan Wang, Zhenting Huang, Feng Jin, Qian Zou, Jing Li, Yinshuang Pu, Zhiyou Cai

**Affiliations:** ^1^Department of Neurology, Chongqing General Hospital, University of Chinese Academy of Sciences, Chongqing, 400013, Chongqing, China.; ^2^Chongqing School, University of Chinese Academy of Sciences, Chongqing, China.; ^3^Chongqing Key Laboratory of Neurodegenerative Diseases, Chongqing, 400013, Chongqing, China.; ^4^Chongqing Medical University, Chongqing, China.

**Keywords:** aging, neuro-glia-vascular unit, energy metabolism

## Abstract

An emerging concept termed the “neuro-glia-vascular unit” (NGVU) has been established in recent years to understand the complicated mechanism of multicellular interactions among vascular cells, glial cells, and neurons. It has been proverbially reported that the NGVU is significantly associated with neurodegenerative disorders, such as Alzheimer’s disease (AD), Parkinson’s disease (PD), and amyotrophic lateral sclerosis (ALS). Physiological aging is an inevitable progression associated with oxidative damage, bioenergetic alterations, mitochondrial dysfunction, and neuroinflammation, which is partially similar to the pathology of AD. Thus, senescence is regarded as the background for the development of neurodegenerative diseases. With the exacerbation of global aging, senescence is an increasingly serious problem in the medical field. In this review, the coupling of each component, including neurons, glial cells, and vascular cells, in the NGVU is described in detail. Then, various mechanisms of age-dependent impairment in each part of the NGVU are discussed. Moreover, the potential bioenergetic alterations between different cell types in the NGVU are highlighted, which seems to be an emerging physiopathology associated with the aged brain. Bioenergetic intervention in the NGVU may be a new direction for studies on delaying or diminishing aging in the future.

Aging is a complex and irreversible process characterized by a gradual loss of biological function and increased susceptibility to neurodegenerative diseases [1]. According to *World Population Aging*, in 2019, the world’s population over 65 years of age had reached 703 million, which is approximately 9% of the world's population [2]. It is estimated that the global aging population is expected to increase to 1.5 billion by 2050 or approximately 16% of the world’s population [2]. It is worth mentioning that the underlying cellular and molecular changes in the aging brain, including oxidative stress, mitochondrial damage, altered glucose metabolism, and neuroinflammation, are strikingly similar to the pathological changes in Alzheimer’s disease (AD) [3].

The brain accounts for only 2% of the total body weight, but consumes approximately 20% of the total oxygen and 25% of the total glucose [4]. Most glucose expended in the brain is mainly used to transduce energy through glycolysis and mitochondrial oxidative phosphorylation to maintain and complete synaptic transmission [5]. However, glucose utilization in the human brain is significantly reduced during aging [6] and is associated with reduced insulin-sensitive glucose transporter expression [7]. Low glucose utilization leads to tau hyperphosphorylation [8] and synaptic dysfunction [9], which are the core pathological features of AD. These data imply that brain senescence may be a precursor of AD.

The neuro-glia-vascular unit (NGVU) is an emerging concept defined by Harder [10] that emphasizes neurovascular coupling among neurons, glial cells, and vascular cells. Each component of the NGVU cooperates with each other to maintain brain homeostasis under physiological conditions [11]. However, recent studies have shown that physiological aging may lead to damage or dysfunction of NGVU components [12], which acts as a background for neurodegenerative diseases. In this review, the various components of the NGVU and their structural and functional coupling with each other are first described. Moreover, we discuss the role of NGVU dysfunction in neurodegenerative disorders such as AD, PD, and ALS. In addition, age-related impairments in the morphology and biological function of NGVU components are elucidated in detail. Furthermore, this work highlights the precise mechanism of energy metabolism dysfunction between different types of cells (such as between neurons and astrocytes or neurons and oligodendrocytes) in the NGVU during aging.

**Figure 1.**
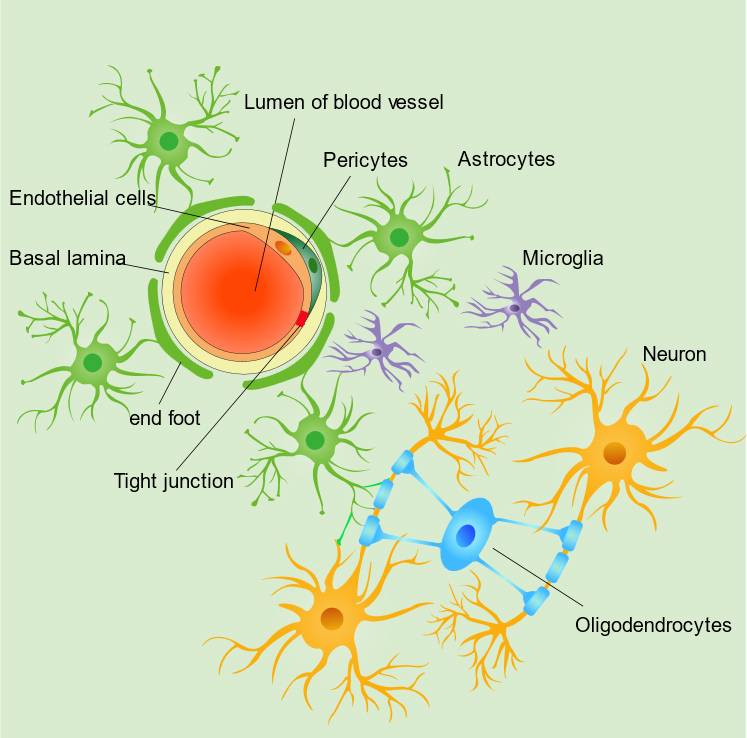
Structure schematic of neuro-glia-vascular unit.

## 1.Major components of the NGVU

Dysfunction after brain injury or disease is mainly caused by neuronal damage. Over the past few years, the concept of the NGVU has been proposed to understand the progression of cerebral ischemic injury and neurodegenerative diseases [13, 14]. This emerging concept highlighted the interactions among multiple cell types but did not focus on identifying the unique contribution of each cell type, which has revised the neuro-centric view of brain diseases. The NGVU is a structural and functional unit composed of neurons, glial cells (oligodendrocytes, microglia, and astrocytes), vascular cells (endothelial cells (ECs), pericytes, and smooth muscle cells (SMCs)), and the basal lamina [15, 16] (Fig. 1). The functional coupling of these diverse components of the NGVU is essential for neurovascular coupling, brain metabolism dynamics, and the maintenance of neuronal homeostasis [17]. Furthermore, the NGVU plays a vital role in the production and development of the blood brain barrier (BBB) to maintain a stable environment for brain function [18, 19].

### 1.1 Glial cells in the NGVU

Glial cells, including astrocytes, microglia, and oligodendrocytes, perform specific functions to maintain central nervous system (CNS) homeostasis. Astrocytes are the most abundant cells in the human brain and serve as a bridge to build connections between neurons and cerebral microvessels [20, 21]. In fact, astrocytes play important roles in maintaining the metabolic and ion homeostasis of neuronal cells, modulating synaptic transmission, and spreading glutamate-induced excitatory signals [22-24]. Because there is almost no direct contact between neurons and microvessels, some essential materials, such as glucose and oxygen supplied from the cerebral circulation, must interact with astroglia before reaching the neurons [25]. Additionally, astrocytes control BBB permeability by stretching their end-feet to the microvessel to interact with ECs [26]. Within the NGVU, microglia act as the first defense in protecting the CNS from pathogen infringement and clearing cell debris [27]. The interaction between microglia and astrocytes leads to neuroinflammation cascade amplification [28, 29]. As different parts of the NGVU, microglia and astrocytes enhance immunological functions through their synergy to build a cascade amplification of neural networks [30]. Oligodendrocytes produce lipid-enriched myelin to ensheath axons of the neurons and expedite the conduction of nerve impulses [31].

### 1.2 Vascular cells in the NGVU

The vascular components of the NGVU mainly include ECs, pericytes, and SMCs [13]. ECs, the typical and central part of the NGVU, are the major structural elements of the BBB, which are regulated by adjacent pericytes, astrocytes, and neurons [15]. Recent studies have shown that pericytes display various functions in the NGVU, such as maintenance of BBB integrity through interaction with ECs and astrocytes [32-34], phagocytosis and immunological functions [35], blood flow control [36], and stem cell function [37]. Furthermore, ECs, pericytes, and astrocytes interact with each other to form tight junctions, which regulate BBB permeability in the NGVU [38]. Vascular smooth muscle cells in the NGVU contribute to the modulation of microvascular blood flow in the brain [36]. However, an in-depth understanding of the precise function of pericytes and SMCs is warranted. Additionally, the extracellular matrix, which is produced by both ECs and pericytes, forms the basal lamina of capillaries in the CNS [39].

### 1.3 Neurons in the NGVU

Neurons, the basic units constituting the structure and function of the nervous system, serve a variety of functions, including stimulation, integration of information, impulse conduction, and adjustment of BBB function [40]. Neurons detect subtle changes in glucose and oxygen and transmit unknown electrical and chemical signals, such as glutamate signaling to astrocytes [41, 42]. As a bridge connecting neurons and microvessels, astrocytes play a vital role in the regulation of essential materials in neurons [43]. One side of astroglial end-feet combined with presynaptic and postsynaptic neurons is called a tripartite synapse [44, 45]. Furthermore, almost all microvessel surfaces are covered by astroglial end-feet, and astrocytes are connected to each other via gap junctions with connexin 43 channels, forming an overall functional syncytium [46]. This evidence indicates that neurovascular communication occurs through astroglial end-feet. However, the precise mechanism by which the supply of nutritious materials from brain capillaries is provided to neurons remains unclear.

## 2. NGVU in age-related neurodegenerative diseases

### 2.1 NGVU and AD

AD, as a progressive neurodegenerative disease, is clinically characterized by extensive memory loss and continuous cognitive dysfunction [47]. Insoluble Aβ deposition in senile plaques is widely known to be the key pathological mechanism of AD [48]. With extensive research, dysfunction of the aged NGVU is considered to be highly related to Aβ accumulation [49]. According to the two-hit hypothesis of AD from Zlokovic [50], NGVU dysfunction occurs before the disruption of Aβ homeostasis in the CNS. Under normal circumstances, peripheral Aβ is prevented from entering the brain by the intact BBB, and Aβ in the CNS is cleared in time [51]. However, as aging proceeds, decreased expression of Aβ efflux transporters (LRP1 and P-gp) on the endothelium [52, 53] and increased expression of Aβ influx transporters (RAGEs) [54] could lead to the accumulation of Aβ in the aged brain, which finally accelerates the progression of AD.

In addition to Aβ accumulation, neuroinflammation plays a fundamental role in AD pathology [55]. Increased leakage is observed in the BBB during aging [56], which indicates that more substances may leak into the brain and abnormally activate microglia, inducing neuro-inflammation [57]. Microglia, as defensive cells in the NGVU, have prominent phagocytic functions to clear invading pathogens, abnormal proteins, and residual debris from the CNS [58]. With advanced aging, microglia show declined capacity of self-renewal [59], reduced motility [60], non-homogeneous distribution [61], and a diminished ability to internalize Aβ peptides [58], which implies the disrupted clearance of Aβ and a more toxic environment for neurons.

### 2.2 NGVU and PD

PD is a progressive and incurable neurodegenerative disorder with motor and non-motor symptoms [62]. Motor symptoms, such as quiescent tremor, bradykinesia, myotonia, and postural gait disorders, can be preceded by non-motor symptoms that include depression, constipation, and sleep disorders [63]. The loss of substantia nigra pars compacta dopaminergic neurons is the hallmark of PD pathogenesis. Dopaminergic neurons contain abundant mitochondria due to their high-metabolic demand and have high levels of reactive oxygen species (ROS) [64]. In senescent cells, mitophagy is impaired [65] and energy support declines, which lead to reduced mitochondria repair, finally resulting in dopaminergic neuronal loss [66].

Addition to neural injury, the disturbance of glial cells in the NGVU contribute to the progression of PD. Physiologically, astrocytes protect dopaminergic neurons from the invasion of toxic molecules, including glutamate and α-synuclein, by producing antioxidants [67]. However, the phenotype of astrocytes is altered [20] and microglia are activated by chronic inflammation in the aged brain [58]. Moreover, senescent astrocytes and microglia produce various cytokines, chemokines (such as TNF-α, IL-1β, and IL-6), and ROS, which damage the integrity of the NGVU [68, 69]. As described above, P-gp on the BBB is decreased with aging. It has been reported that decreased P-gp may be related to the accumulation of α-synuclein in the brain [70]. Misfolded α-synuclein aggregates that form Lewy bodies and neurites are confirmed to be the histological hallmark of PD [62]. Obviously, aging is a risk factor for the development of neurodegenerative diseases. Although the crucial mechanisms remain unclear, age-related impairment of the NGVU partly contributes to the initiation and maintenance of the malignant cycle in neurodegeneration. Thus, targeting aged NGVU to prevent the progression of neurodegenerative disorders may be a highly feasible therapeutic strategy.

## 3. Impact of senescence on the components of the NGVU (Table 1)

### 3.1 Age-related effect on neurons

During the aging process, nuclear DNA lesions occur extensively in neurons; proteins involved in DNA methylation and acetylation, DNA repair, and lipid metabolism are disrupted over time with aging [71]. Moreover, DNA repair dysfunction exacerbates DNA damage [72]. Furthermore, these age-related nuclear DNA lesions cause mitochondrial DNA damage, resulting in mitochondrial dysfunction [72]. In addition to nuclear DNA lesions, altered protein modifications, such as carbonylation, nitration, and covalent binding of the lipid peroxidation product 4-hydroxynonenal, during aging lead to reduced activity of various proteins, which fail to prevent toxic protein aggregation and oxidative stress [12].

Age-related impairment of the mitochondria and endoplasmic reticulum (ER) plays an important role in neuronal functional decline [73-76]. The damaged mitochondria and ER lose the ability to sequester Ca^2+^, which leads to increased concentrations of intracellular Ca^2+^, inducing ROS production, resulting in neuronal apoptosis [71, 77]. Meanwhile, decreased ROS clearance with aging enhances oxidative stress and neuronal apoptosis [71]. Increasing evidence has shown that the ER, performing functions in synthesis and the modification and processing of proteins, is distinctly impaired with aging [78, 79]. With advancing age, ER-related stress significantly increases [80] and ER-related chaperones, such as glucose regulatory protein 78, decline in the brain, which is associated with the progression of neurodegeneration [81]. Additionally, deterioration of synaptic plasticity and deficits in long-term potentiation occur in the aged brain, which may partly be caused by increased activation of microglia [82].

During normal aging, the total number of neurons shows no significant change in the hippocampus and many regions of the cortex. However, a massive loss of principal neurons in the dorsal primate prefrontal cortex was reported in a recent study [83]. This change implies that the loss of neurons in the aged brain may be regional. Most neurons in the CNS are non-renewable, except in some specific areas, such as the subventricular zone (SVZ) and subgranular zone (SGZ) of the dentate gyrus [84, 85]. However, senescence reduces neuronal regeneration capacity in the SVZ and SGZ [86, 87]. β2-microglobulin and insulin-like growth factor-1 are negative regulators of neurogenesis associated with aging [86, 88]. During aging, negative regulation decreases, leading to excessive activation of glutamate (a major excitatory neurotransmitter in the CNS) and subsequent excitotoxicity [89]. Moreover, as discussed above, maladjustment of Ca^2+^ regulation causes neuronal vulnerability to excitotoxicity, further accelerating the progress of neuronal apoptosis [71].

### 3.2 Age-related effect on glial cells in the NGVU

#### 3.2.1 Astrocytes

As a multifunctional cell type in the NGVU, astrocytes show senescence-related changes in number, morphology, and function during aging; in a study by Harris [90], the number and size of astrocytes increased significantly with aging. However, a different view from Rodriguez-Arellano [91] and Vartak-Sharma [92] indicated that the number of astrocytes does not change dramatically in the aged brain. In addition, the expression of glial fibrillary acidic protein increases progressively in the elderly compared to that in the young [93-96], which has been demonstrated to be highly linked to a flat, senescent morphology of astrocytes [20, 97]. Furthermore, the concentration of glutamate transporters, glutamate-aspartate transporter, and glutamine synthase are decreased in senescent astrocytes [98-100]; these alterations lead to the dysfunction of glutamate regulation, which contributes to neuronal excitotoxicity. The function of aquaporin 4 in aged astrocytes is also upregulated [101], which leads to diminished clearance of CNS metabolites. Neurotransmitter-induced Ca^2+^ signaling is reduced in aged astrocytes, which affects the release of neuro-active substances that support neuronal functions [20, 102].

In a review by Antero [20], it was reported that oxidative stress, proteotoxic aggregation, and metabolic stress persist in the aging brain. Oxidative stress and proteotoxic aggregation induce astrocytes to secrete proinflammatory cytokines, such as interleukin-6 (IL-6) and metalloproteinase (MMP)-9, resulting in inflammation in the aging brain [20]. Therefore, senescent astrocytes are also identified as “A1” reactive astrocytes (astrocytes of a more inflammatory state) [103], which secrete a neurotoxin that kills neurons [29]. Meanwhile, the metabolic stress of astrocytes in the senile brain reduces the capacity of astrocytes to support metabolism transportation to neurons [104]. Thus, astrocytes develop a poor supportive function and exhibit increased secretion of harmful factors with increasing age, which, to a large extent, affects the survival of neurons.

#### 3.2.2 Microglia

During aging, microglia exhibit retrogressive changes in different aspects; compared to young microglia, aged microglia become much less motile with the supply of adenosine triphosphate (ATP), which indicates a lower patrol capability in the NGVU [105]. Abnormal phenotypic shifts are detected in aged microglia [106-108]. Senescent microglia tend to polarize to the M1 phenotype with specific morphological features, including increased soma volume and thicker processes [105]. Meanwhile, microglia in the aged brain are evenly distributed compared with those in the young brain [61], which may be because of altered morphology or an increased number of microglia [109]. Moreover, elevated expression of proinflammatory markers, such as major histocompatibility complex (MHC)-II, CD16/32, CD86, interferon-γ, inducible nitric oxide synthase, interleukin-1β, IL-6, tumor necrosis factor-α, and lipofuscin granules are also found in aged microglia [27, 73]. Several studies [110, 111] have suggested that regulatory factors, such as CD200, C-X3-C motif chemokine ligand 1, and gamma-aminobutyric acid from neurons and nuclear factor erythroid 2-related factor 2, transforming growth factor-β, macrophage colony-stimulating factor, and granulocyte/macrophage colony stimulating factor from astrocytes could play a key role in the shift in microglia towards an M1 phenotype during aging. However, the precise mechanism of such detrimental phenotypic shifts in senescent microglia requires further in-depth research. Increasing evidence implies that there are more complex and active interactions between glial cells and neurons.

Palmer et al. [112] have hypothesized that both astrocytes and microglia may be involved in the elimination of neurons during aging. Previous work from different laboratories has suggested that senescent microglia increase the reactivity of astrocytes through their secretory profile [29, 113, 114].

#### 3.2.3 Oligodendrocytes

In the CNS, oligodendrocytes wrap axons and form insulating myelin, playing a vital role in efficient nerve conduction. Oligodendrocytes have swelling morphology in the aging brain caused by degenerated inclusions of myelin sheaths in the cytoplasm [115]. Additionally, myelin formed by senescent oligodendrocytes is thinner with shorter internode lengths, which results in a reduced ability of myelin to increase nerve conduction velocity [116]. Moreover, the differentiation [117, 118] and recruitment [118, 119] of oligodendrocyte precursor cells, as replacements for oligodendrocytes in the mature CNS, are impaired during the aging process; these alterations may lead to a decrease in oligodendrocytes. Additionally, oligodendrocytes are associated with the metabolism and nutrition of axons and decreased oligodendrocytes may cause deterioration and death of the axon [120, 121].

### 3.3 Age-related effects on microvascular components in the NGVU

#### 3.3.1 ECs

Cerebral ECs are the main component of the BBB and play various roles in the NGVU [122]. The number of ECs was found to be decreased in the aged brain [123]. The loss of ECs may lead to the morphological elongation and thinning of remaining ECs [123]. The function of ECs is closely linked to the mitochondria; during physiological aging, the number and function of the mitochondria are significantly decreased [124], which is damaging to ECs [50]. Nitric oxide (NO), the most important endothelium-derived vasodilator for ECs, exerts powerful anti-inflammation, anti-apoptosis, and pro-angiogenesis effects and modulates cellular metabolism, mitochondrial function, and synaptic transmission [125-129]. Senescence leads to increased mitochondrial production of superoxide [130], which reacts with NO to form peroxynitrite, resulting in reduced availability of NO and endothelial dysfunction [131-133]. The activity and expression of endothelial NO synthase is reduced during advanced aging [134, 135], which directly inhibits the production of NO [136]. Thus, an age-related reduction in the production of endothelium-derived NO negatively affects the function of microvessels, neurons, astrocytes, and microglia.

In the context of aging, the reduced regeneration capacity and newly generated defective ECs are irreversible changes involving multiple mechanisms [137-139]. First, the secretion of growth factors, such as vascular endothelial growth factor, is impaired, resulting in the growth arrest of ECs [139]. Moreover, elevated levels of tissue inhibitors of metalloproteinase during aging inhibit the expression of MMP-9, which plays a vital role in angiogenesis [138]. In a review by Gradinaru [137], newborn ECs were reported to show functional defects with aging, including increased low-density lipoprotein and reduced NO production.

**Table 1:** Alterations of NGVU constituents during physiological aging.

NGVU constituent	Alterations during physiological aging	References
Neurons	Age-related accumulation of nuclear DNA damage was found to be in 12-month Han/NMRI mice.	[71, 72]
	Declined function of mitochondrion in aging rats were widely reported in several studies.	[74-76]
	The number of DCX+ newly born neurons decrease progressively with increasing age (from 7.5 months to 12 months) in the subgranular zone in F344 rats	[87]
Astrocytes	Astrocytes presented a flat, senescent morphology with aging in Hwang’s study.	[97]
	The expression of GFAP is significantly increased in the hippocampus of human brain.	[93, 95, 96]
	An age-dependent decrease for GLAST and GS expression levels were observed in aged astrocytes from Wistar rats or Sprague-Dawley rats.	[99, 100]
	In vivo, age-related over-abundance in Ca^2+^ was associated with an increase in JNK/SAPK activation, which has been linked to cell death signaling.	[102]
	Senescence astrocytes affects aging brain though oxidative stress, proteotoxic aggregation, metabolic stress and inflammation.	[20]
Microglia	Microglia from aged C57BL/6 mice showed a reduced patrol capability	[105]
	Age-dependent switch from the alternative M2 to the classical M1 phenotype was detected in APP/PS1 mice hippocampus at age of 18 months.	[108]
	Senescent microglia express proinflammatory markers including MHC-II, IL-1β, IL6, TNF-α and lipofuscin granules.	[27, 73]
Oligodendrocytes	Oligodendrocytes from a 35-year-old rhesus monkey showed a swelling morphology along their lengths.	[115]
	OPCs showed declined capacity of differentiation and recruitment in female Sprague Dawley rats. The recruitment of progenitor cells to replace lost OLs was impaired in C57BL/6 mice.	[118, 119]
Endothelial cells	MnSOD (manganese superoxide dismutase) in endothelial cells from aged Fischer-344 rats were decreased significantly, which indicates that mitochondrial dysfunction could be an important mediator of vascular lesions.	[124]
	Reduced production of endothelium-derived NO was found in Fischer-344 rats. Deficiency in eNOS substrates (L-arginine, in human) and the presence of endogenous eNOS inhibitors (symmetric dimethylarginine, in human) in advancing age result in lower expression of eNOS.	[133-135]
	Reduced capacity of regeneration and newly generated defective endothelial cells are also characteristic alterations in aged brain.	[137, 139]
Pericytes	Ultrastructural alterations including vesicular and lipofuscin-like inclusions, increased size of mitochondria and foamy conversion in senescent pericytes were reported in several studies.	[140-145]
	The loss of pericytes are reported in the brain of aged male Wistar rats and human.	[146, 147]
	No change in the number of pericytes in the brain of 35-year rhesus monkeys. Pericyte populations are increased in the 36-month rat parietal cortex.	[142, 145]
	Age-dependent diminished neurotrophic support was come forward in Bell’s study.	[148]

#### 3.3.2 Pericytes and SMCs

Pericytes, as specialized SMCs, display ultrastructural alterations, such as vesicular and lipofuscin-like inclusions, increased mitochondrial size, and foamy conversion, with physiological aging [140-145]. The number of pericytes is decreased in the brains of both aged rats and humans [146, 147]. Robert et al. [148] indicated that pericyte deficiency leads to a reduction in brain microcirculation and BBB breakdown associated with brain accumulation of several toxic protein molecules. However, different studies have shown no change in the number of pericytes in monkeys [142] and increased pericytes in the aging rat brain [145, 149]. These discrepancies may be associated with distinct mechanisms of age-related pericyte degeneration. In addition, the secretion of various neural trophic factors is disturbed in aged pericytes [148], and senescent SMCs induced by H_2_O_2_ have a proinflammatory role through the upregulation of IL-6, chemokines, and innate immune receptors [150]. Previous studies have attempted to explore the precise mechanisms of age-related changes in pericytes and SMCs; however, more evidence is required to identify the role of senescent pericytes in the structural and pathological dysfunctions of the NGVU.

**Figure 2.**
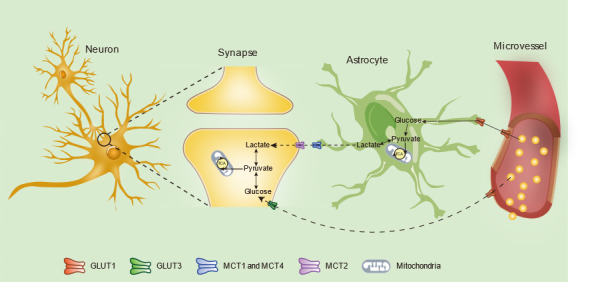
Glucose metabolism between microvessel, astrocyte and neuron in the neuro-glia-vascular unit. Glucose supplied from microvessels can be directly transported to astrocytes by GLUT1(Glucose transporter 1) and neurons by GLUT1 and GLUT3(Glucose transporter 3). Glucose transported into astrocytes produces lactate via glycolytic pathway. Then, lactate, served as an energetic substrate for neurons, is transported to neurons through MCT1 (Monocarboxylate transporter 1) and MCT4 (Monocarboxylate transporter 4) (astrocytic form) and MCT2 (neural form).

## 4. Altered connection of energy metabolism in the NGVU during advanced age

### 4.1 Impaired energy metabolism between astrocytes and neurons

Neurons are indispensable in the CNS, as well as in the NGVU. In the past two decades, numerous studies have suggested that the energy metabolism of astrocytes is a key factor supporting the high energy requirement of neurons [43, 151-153]. Metabolic interactions between astrocytes and neurons include three components, 1) glucose and lactate, 2) fatty acids and ketone bodies (KBs), and 3) D- and L-serine. Among these components, glucose metabolism seems to be the foremost pathway for energy metabolism between astrocytes and neurons (Fig. 2). Previous data show that astrocytes cultured *in vitro* produce large amounts of lactate and neurons produce less lactate from glucose under normal (21%) oxygen environments [154], which implies that astrocytes take up glucose from microvessels and produce lactate as a supplement for neurons. Under normal circumstances, glucose is transported into astrocytes through GLUT1, and astrocytes generate ATP and lactate through anaerobic glycolysis [43]. Subsequently, lactate is released from astrocytes and is taken up by neighboring neurons through monocarboxylate transporters (MCTs), which is called the astrocyte-neuron lactate shuttle (ANLS) [153]. Except for the mediation of astrocytes, GLUT3 allows the direct import of glucose from blood vessels into neurons [151]. However, astrocytes are likely to be responsible for most energy supplementation to neurons because 99% of the microvessel surface is covered by astrocyte end-feet [25, 151, 155]. Moreover, a study on GLUT3-knockout mice demonstrates that mice survive without glucose when lactate is available [156].

**Figure 3.**
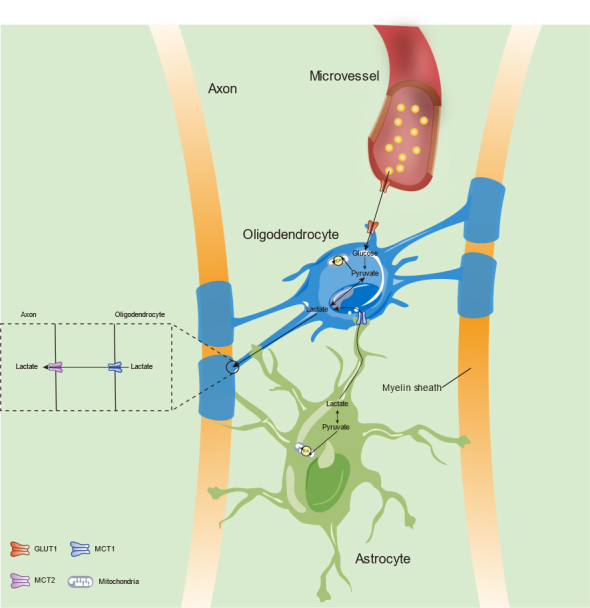
Mechanisms of energy metabolism in the oligodendrocytes. Oligodendrocytes have continuous energic requirement to support the function of axons and the form of myelin. Glucose is taken up from microvessels through GLUT1 and produces lactate via glycolysis. Also, lactate can be taken up from astrocytes through MCT1. Then, lactate in oligodendrocytes is transported to axon through MCT1 (form in oligodendrocytes) and MCT2 (neural form).

GLUT1 is an important mediator for the passage of glucose through the blood into astrocytes, which indirectly provides energy to neurons [43]. In a study by Souza [157], aged astrocytes showed decreased GLUT1 expression compared to that of newborn astrocytes, indicating reduced transportation of glucose from blood into astrocytes. Moreover, Fan et al. have reported significantly decreased GLUT1 expression and age-related declines in GLUT3 expression in 15-month-old mice [158]. In contrast, an age-related decrease in the expression of GLUT3 was found in the hippocampus of 29-month-old Wistar rats [159]. Furthermore, MCT1, a key mediator of the ANLS, declines with aging [158]. Reduced expression of multiple transporters in the energy transport pathway from astrocytes to neurons might be an important cause of neuronal metabolic disorders during aging. Recently, an imaging study has showed decreased neuronal mitochondrial metabolism and increased glial mitochondrial metabolism in the elderly compared to those in young people [160]. Upregulated mitochondrial metabolism in astrocytes may be an energy-saving way to meet energy demands under stress. Additionally, one study on primary cortical astrocytes isolated from rats of different ages indicated that the metabolic shift in astrocytes from anaerobic respiration towards mitochondrial metabolism is positively associated with increasing age [161]; this metabolic shift leads to decreased production of lactate, which hampers the ANLS and deprives neurons of their energy supply. Overall, enhanced mitochondrial metabolism and decreased lactate levels may partly explain the state of metabolic insufficiency in the senile brain.

### 4.2 Reduced bioenergetics between oligodendrocytes and neurons

Oligodendrocytes have high energy demands to form myelin and interact with axons. Recent studies suggest that oligodendrocytes take up lactate from astrocytes through MCT1 and glucose from microvessels through GLUT1 [162] (Fig. 3). Lactate may then be shuttled to and taken up by axons through MCT1 on oligodendrocytes and MCT2 on neurons; several studies have indicated that glucose and lactate seem to be substrates for oligodendrocytes to support the energy metabolism of axons [121, 163-165].

During normal aging, the abnormal ultrastructure of myelin in the CNS potentially reduces normal axon-glia metabolic coupling [166]. As discussed previously, the expression of MCT1 is reduced during aging [158], which may affect the energy supply from oligodendrocytes to axons. In addition, MCT1-knockout oligodendrocytes in transgenic mice have decreased metabolism and axonopathy [121]. On the basis of a previous study that showed decreased expression of GLUT1 in senescent astrocytes [157], we hypothesized that impaired energy transport from astrocytes to oligodendrocytes may indirectly influence the metabolism between oligodendrocytes and axons. Nevertheless, the specific mechanism of altered bioenergetics between oligodendrocytes and neurons during physiological aging requires more *in vitro* and *in vivo* studies.

### 4.3 Senescence-related energy metabolism of microglia in the NGVU

Glucose, fatty acids, and glutamine are reported to be energy substrates for microglia [167]; among these substrates, glucose seems to be the main source of energy. Like neurons, microglia express GLUT3, which directly transports glucose from brain vessels into microglia [168]. In addition, microglia express other GLUTs under different circumstances; interestingly, GLUT5, a passive fructose transporter, is only expressed in microglia in the brain [169]. However, the precise function of GLUT5 in microglia remains unclear.

A great deal of evidence suggests that aged microglia are associated with a more reactive/activated phenotype in both human and rat brains [109, 170, 171]. MHC II antigens, CD11b, CD14, and pattern recognition receptors, which are expressed in activated microglia, are also elevated in senescent microglia [172-175]. In addition, considering the reduced glucose metabolism during aging [176], astroglia may be activated in this environment [167, 171]. Furthermore, healthy neurons and astrocytes maintain microglia in a resting state with low energy demands [177, 178]. However, physiologically aged neurons and astrocytes may partly contribute to the activation of microglia. This evidence may support the view that more quiescent microglia are activated during aging. Since activated microglia constantly require ATP and the availability of glucose in the brain is not infinite, astroglia may take up energy from the other two energy substrates (fatty acid and glutamine) when glucose is deficient [168]. The data presented in a study by Antwoine et al. [179] suggest that aged microglia exhibit a bioenergetic shift from glucose to fatty acid utilization, which indicates energy metabolism dysfunction in senescent microglia. However, there is no powerful evidence to explain the relationship between declined metabolism in aged microglia and the NGVU. Nevertheless, as patrol and defensive cells in the NGVU, astroglia are undoubtedly impaired in various functional ways caused by bioenergetic disorders during advanced age.

### 4.4 Decreased energy supply from brain microvascular ECs to astrocytes and neurons

Glucose must be continuously supplied to the NGVU via blood circulation due to the lack of glucose in the brain [180, 181]. Energetic substrates are delivered from the blood to astrocytes or neurons by several types of transporters [151, 182]. GLUT1 and GLUT3, two key glucose transporters in the NGVU, are decreased in ECs during aging [157-159, 183], implicating age-related inefficiency of glucose supply from blood to the NGVU. Apart from the reduced expression of GLUTs, the dysfunction of the GLUT1 transporter transferring glucose from the blood to the brain may partly contribute to this inefficient energy transport in the senescent NGVU [184, 185]. In addition to glucose, KBs are believed to be a better source from blood to neurons during ischemia or hypoxia [43]. The KB used for the brain is mostly produced in the liver and transported from blood to astrocytes, as well as to some neurons, by MCT1 on ECs [186]. In the hippocampus of female mice, MCT1 expression shows a decreasing trend from 9 to 15 months of age [158], suggesting an impaired capacity of KB transportation from ECs to neurons with aging. Nevertheless, the data on age-related modulation of the expression of MCT1 are lacking and require more targeted studies to verify this in the future.

## 5. Conclusions and Perspectives

Population aging is becoming a worldwide issue that severely influences social and economic development. With a deeper understanding of the concept of the NGVU, age-related impairment of the NGVU has gradually appeared as a characteristic change during physiological aging and an increasing risk factor for neurodegenerative disorders. Considering the close cooperation between each component in the NGVU, the damage caused by senescence is disastrous for the morphology and function of neurons, glial cells, and vascular cells. Furthermore, the bioenergetic metabolism cascade between different cell types in the NGVU is altered with advanced age. Decreased transportation capability of energetic substrates involved with GLUTs and MCTs and an age-related metabolic shift may be responsible for the impairment of bioenergetic metabolism in the NGVU. Nevertheless, the precise mechanism remains an enormous challenge for present academic standards due to the intricate and subtle changes in energy metabolism shuttling in different cell types. Senescent bioenergetic alterations in the NGVU are expected to be a newly developed physiopathology of the aged brain. Thus, bioenergetic intervention for the brain may be a viable and practical therapy to prevent and delay the process of aging in prospective medical fields.

## References

[1] Pan Y, Nicolazzo JA (2018). Impact of aging, Alzheimer's disease and Parkinson's disease on the blood-brain barrier transport of therapeutics. Advanced Drug Delivery Reviews, 135:62-74. 29665383 10.1016/j.addr.2018.04.009

[2] <1.World Population Ageing 2019_ Highlights.pdf>.

[3] Poddar J, Pradhan M, Ganguly G, Chakrabarti S (2019). Biochemical deficits and cognitive decline in brain aging: Intervention by dietary supplements. J Chem Neuroanat, 95:70-80. 29678666 10.1016/j.jchemneu.2018.04.002

[4] Takahashi S, Abe T, Izawa Y, Suzuki N (2012). Effects of fluctuating glucose concentrations on oxidative metabolism of glucose in cultured neurons and astroglia. Journal of Diabetes Mellitus, 02:19-26.

[5] Lewis S (2016). Synaptic transmission: Go, go, glycolysis! Nature reviews. Neuroscience, 17:334-335. 10.1038/nrn.2016.55 27150402

[6] Briones TL, Darwish H (2014). Decrease in age-related tau hyperphosphorylation and cognitive improvement following vitamin D supplementation are associated with modulation of brain energy metabolism and redox state. Neuroscience, 262:143-155. 24412233 10.1016/j.neuroscience.2013.12.064 PMC4103183

[7] Taguchi A, Wartschow LM, White MF (2007). Brain IRS2 signaling coordinates life span and nutrient homeostasis. Science, 317:369-372. 17641201 10.1126/science.1142179

[8] Kapogiannis D, Mattson MP (2011). Disrupted energy metabolism and neuronal circuit dysfunction in cognitive impairment and Alzheimer's disease. The Lancet Neurology, 10:187-198. 21147038 10.1016/S1474-4422(10)70277-5 PMC3026092

[9] Butterfield DA, Halliwell B (2019). Oxidative stress, dysfunctional glucose metabolism and Alzheimer disease. Nat Rev Neurosci, 20:148-160. 30737462 10.1038/s41583-019-0132-6 PMC9382875

[10] Harder D, Zhang C, Gebremedhin D (2002). Astrocytes function in matching blood flow to metabolic activity. News in physiological sciences : an international journal of physiology produced jointly by the International Union of Physiological Sciences and the American Physiological Society, 17:27-31. 10.1152/physiologyonline.2002.17.1.27 11821533

[11] Muoio V, Persson PB, Sendeski MM (2014). The neurovascular unit - concept review. Acta Physiol (Oxf), 210:790-798. 24629161 10.1111/apha.12250

[12] Cai W, Zhang K, Li P, Zhu L, Xu J, Yang B, et al. (2017). Dysfunction of the neurovascular unit in ischemic stroke and neurodegenerative diseases: An aging effect. Ageing Res Rev, 34:77-87. 27697546 10.1016/j.arr.2016.09.006 PMC5384332

[13] Yan C, Zhou Y, Chen Q, Luo Y, Zhang JH, Huang H, et al. (2020). Dysfunction of the neurovascular unit in diabetes-related neurodegeneration. Biomed Pharmacother, 131:110656. 32841897 10.1016/j.biopha.2020.110656

[14] Zhao Y, Yang J, Li C, Zhou G, Wan H, Ding Z, et al. (2020). Role of the neurovascular unit in the process of cerebral ischemic injury. Pharmacol Res, 160:105103. 32739425 10.1016/j.phrs.2020.105103

[15] Lo EH, Rosenberg GA (2009). The neurovascular unit in health and disease: introduction. Stroke, 40:S2-3. 19064779 10.1161/STROKEAHA.108.534404 PMC2811575

[16] Ting SM, Zhao X, Sun G, Obertas L, Ricote M, Aronowski J (2020). Brain Cleanup as a Potential Target for Poststroke Recovery: The Role of RXR (Retinoic X Receptor) in Phagocytes. Stroke, 51:958-966. 31914884 10.1161/STROKEAHA.119.027315 PMC7042051

[17] Morrison H, Filosa J (2019). Stroke and the neurovascular unit: glial cells, sex differences, and hypertension. American journal of physiology. Cell physiology, 316:C325-C339. 30601672 10.1152/ajpcell.00333.2018 PMC6457101

[18] Bell AH, Miller SL, Castillo-Melendez M, Malhotra A (2019). The Neurovascular Unit: Effects of Brain Insults During the Perinatal Period. Front Neurosci, 13:1452. 32038147 10.3389/fnins.2019.01452 PMC6987380

[19] Sa-Pereira I, Brites D, Brito MA (2012). Neurovascular unit: a focus on pericytes. Mol Neurobiol, 45:327-347. 22371274 10.1007/s12035-012-8244-2

[20] Salminen A, Ojala J, Kaarniranta K, Haapasalo A, Hiltunen M, Soininen H (2011). Astrocytes in the aging brain express characteristics of senescence-associated secretory phenotype. Eur J Neurosci, 34:3-11. 21649759 10.1111/j.1460-9568.2011.07738.x

[21] Zhou Y, Shao A, Yao Y, Tu S, Deng Y, Zhang J (2020). Dual roles of astrocytes in plasticity and reconstruction after traumatic brain injury. Cell Commun Signal, 18:62. 32293472 10.1186/s12964-020-00549-2 PMC7158016

[22] Fellin T (2009). Communication between neurons and astrocytes: relevance to the modulation of synaptic and network activity. J Neurochem, 108:533-544. 19187090 10.1111/j.1471-4159.2008.05830.x

[23] Fiacco TA, Agulhon C, McCarthy KD (2009). Sorting out astrocyte physiology from pharmacology. Annu Rev Pharmacol Toxicol, 49:151-174. 18834310 10.1146/annurev.pharmtox.011008.145602

[24] Verkhratsky A (2010). Physiology of neuronal-glial networking. Neurochem Int, 57:332-343. 20144673 10.1016/j.neuint.2010.02.002

[25] Mathiisen TM, Lehre KP, Danbolt NC, Ottersen OP (2010). The perivascular astroglial sheath provides a complete covering of the brain microvessels: an electron microscopic 3D reconstruction. Glia, 58:1094-1103. 20468051 10.1002/glia.20990

[26] Abbott NJ, Ronnback L, Hansson E (2006). Astrocyte-endothelial interactions at the blood-brain barrier. Nat Rev Neurosci, 7:41-53. 16371949 10.1038/nrn1824

[27] Wolf SA, Boddeke HW, Kettenmann H (2017). Microglia in Physiology and Disease. Annu Rev Physiol, 79:619-643. 27959620 10.1146/annurev-physiol-022516-034406

[28] Kirkley KS, Popichak KA, Afzali MF, Legare ME, Tjalkens RB (2017). Microglia amplify inflammatory activation of astrocytes in manganese neurotoxicity. J Neuroinflammation, 14:99. 28476157 10.1186/s12974-017-0871-0 PMC5418760

[29] Liddelow SA, Guttenplan KA, Clarke LE, Bennett FC, Bohlen CJ, Schirmer L, et al. (2017). Neurotoxic reactive astrocytes are induced by activated microglia. Nature, 541:481-487. 28099414 10.1038/nature21029 PMC5404890

[30] Liu LR, Liu JC, Bao JS, Bai QQ, Wang GQ (2020). Interaction of Microglia and Astrocytes in the Neurovascular Unit. Front Immunol, 11:1024. 32733433 10.3389/fimmu.2020.01024 PMC7362712

[31] Nutma E, van Gent D, Amor S, Peferoen LAN (2020). Astrocyte and Oligodendrocyte Cross-Talk in the Central Nervous System. Cells, 9. 10.3390/cells9030600 PMC7140446 32138223

[32] Gundersen GA, Vindedal GF, Skare O, Nagelhus EA (2014). Evidence that pericytes regulate aquaporin-4 polarization in mouse cortical astrocytes. Brain Struct Funct, 219:2181-2186. 23982198 10.1007/s00429-013-0629-0 PMC4223569

[33] Hill J, Rom S, Ramirez SH, Persidsky Y (2014). Emerging roles of pericytes in the regulation of the neurovascular unit in health and disease. J Neuroimmune Pharmacol, 9:591-605. 25119834 10.1007/s11481-014-9557-x PMC4209199

[34] Liu S, Agalliu D, Yu C, Fisher M (2012). The role of pericytes in blood-brain barrier function and stroke. Current pharmaceutical design, 18:3653-3662. 22574979 10.2174/138161212802002706

[35] Hurtado-Alvarado G, Cabanas-Morales AM, Gomez-Gonzalez B (2014). Pericytes: brain-immune interface modulators. Front Integr Neurosci, 7:80. 24454281 10.3389/fnint.2013.00080 PMC3887314

[36] Quelhas P, Baltazar G, Cairrao E (2019). The Neurovascular Unit: Focus on the Regulation of Arterial Smooth Muscle Cells. Curr Neurovasc Res, 16:502-515. 31738142 10.2174/1567202616666191026122642

[37] Fisher M (2009). Pericyte signaling in the neurovascular unit. Stroke, 40:S13-15. 19064799 10.1161/STROKEAHA.108.533117 PMC2724312

[38] Jo DH, Kim JH, Heo JI, Kim JH, Cho CH (2013). Interaction between pericytes and endothelial cells leads to formation of tight junction in hyaloid vessels. Mol Cells, 36:465-471. 24213675 10.1007/s10059-013-0228-1 PMC3887934

[39] Bergers G, Song S (2005). The role of pericytes in blood-vessel formation and maintenance. Neuro-oncology, 7:452-464. 16212810 10.1215/S1152851705000232 PMC1871727

[40] Hawkins BT, Davis TP (2005). The blood-brain barrier/neurovascular unit in health and disease. Pharmacol Rev, 57:173-185. 15914466 10.1124/pr.57.2.4

[41] Figley CR, Stroman PW (2011). The role(s) of astrocytes and astrocyte activity in neurometabolism, neurovascular coupling, and the production of functional neuroimaging signals. Eur J Neurosci, 33:577-588. 21314846 10.1111/j.1460-9568.2010.07584.x

[42] Filous AR, Silver J (2016). "Targeting astrocytes in CNS injury and disease: A translational research approach". Prog Neurobiol, 144:173-187. 27026202 10.1016/j.pneurobio.2016.03.009 PMC5035184

[43] Takahashi S (2020). Metabolic compartmentalization between astroglia and neurons in physiological and pathophysiological conditions of the neurovascular unit. Neuropathology, 40:121-137. 32037635 10.1111/neup.12639 PMC7187297

[44] Araque A, Parpura V, Sanzgiri R, Haydon P (1999). Tripartite synapses: glia, the unacknowledged partner. Trends in neurosciences, 22:208-215. 10322493 10.1016/s0166-2236(98)01349-6

[45] Eroglu C, Barres BA (2010). Regulation of synaptic connectivity by glia. Nature, 468:223-231. 21068831 10.1038/nature09612 PMC4431554

[46] Giaume C, Koulakoff A, Roux L, Holcman D, Rouach N (2010). Astroglial networks: a step further in neuroglial and gliovascular interactions. Nat Rev Neurosci, 11:87-99. 20087359 10.1038/nrn2757

[47] Huang Y, Mucke L (2012). Alzheimer mechanisms and therapeutic strategies. Cell, 148:1204-1222. 22424230 10.1016/j.cell.2012.02.040 PMC3319071

[48] Vinters HV (2015). Emerging concepts in Alzheimer's disease. Annu Rev Pathol, 10:291-319. 25387055 10.1146/annurev-pathol-020712-163927

[49] Sweeney MD, Kisler K, Montagne A, Toga AW, Zlokovic BV (2018). The role of brain vasculature in neurodegenerative disorders. Nat Neurosci, 21:1318-1331. 30250261 10.1038/s41593-018-0234-x PMC6198802

[50] Zlokovic BV (2011). Neurovascular pathways to neurodegeneration in Alzheimer's disease and other disorders. Nat Rev Neurosci, 12:723-738. 22048062 10.1038/nrn3114 PMC4036520

[51] O'Brien RJ, Wong PC (2011). Amyloid precursor protein processing and Alzheimer's disease. Annu Rev Neurosci, 34:185-204. 21456963 10.1146/annurev-neuro-061010-113613 PMC3174086

[52] Kang D, Pietrzik C, Baum L, Chevallier N, Merriam D, Kounnas M, et al. (2000). Modulation of amyloid beta-protein clearance and Alzheimer's disease susceptibility by the LDL receptor-related protein pathway. The Journal of clinical investigation, 106:1159-1166. 11067868 10.1172/JCI11013 PMC301422

[53] Silverberg G, Messier A, Miller M, Machan J, Majmudar S, Stopa E, et al. (2010). Amyloid efflux transporter expression at the blood-brain barrier declines in normal aging. Journal of neuropathology and experimental neurology, 69:1034-1043. 20838242 10.1097/NEN.0b013e3181f46e25

[54] Silverberg G, Miller M, Messier A, Majmudar S, Machan J, Donahue J, et al. (2010). Amyloid deposition and influx transporter expression at the blood-brain barrier increase in normal aging. Journal of neuropathology and experimental neurology, 69:98-108. 20010299 10.1097/NEN.0b013e3181c8ad2f

[55] Keren-Shaul H, Spinrad A, Weiner A, Matcovitch-Natan O, Dvir-Szternfeld R, Ulland TK, et al. (2017). A Unique Microglia Type Associated with Restricting Development of Alzheimer's Disease. Cell, 169:1276-1290 e1217. 28602351 10.1016/j.cell.2017.05.018

[56] Farrall AJ, Wardlaw JM (2009). Blood-brain barrier: ageing and microvascular disease--systematic review and meta-analysis. Neurobiol Aging, 30:337-352. 17869382 10.1016/j.neurobiolaging.2007.07.015

[57] Gorle N, Van Cauwenberghe C, Libert C, Vandenbroucke RE (2016). The effect of aging on brain barriers and the consequences for Alzheimer's disease development. Mamm Genome, 27:407-420. 27143113 10.1007/s00335-016-9637-8

[58] Harry GJ (2013). Microglia during development and aging. Pharmacol Ther, 139:313-326. 23644076 10.1016/j.pharmthera.2013.04.013 PMC3737416

[59] Ojo J, Rezaie P, Gabbott P, Stewart M (2015). Impact of age-related neuroglial cell responses on hippocampal deterioration. Frontiers in aging neuroscience, 7:57. 25972808 10.3389/fnagi.2015.00057 PMC4413780

[60] Damani M, Zhao L, Fontainhas A, Amaral J, Fariss R, Wong W (2011). Age-related alterations in the dynamic behavior of microglia. Aging cell, 10:263-276. 21108733 10.1111/j.1474-9726.2010.00660.x PMC3056927

[61] Tremblay ME, Zettel ML, Ison JR, Allen PD, Majewska AK (2012). Effects of aging and sensory loss on glial cells in mouse visual and auditory cortices. Glia, 60:541-558. 22223464 10.1002/glia.22287 PMC3276747

[62] Klingelhoefer L, Reichmann H (2015). Pathogenesis of Parkinson disease--the gut-brain axis and environmental factors. Nature reviews. Neurology, 11:625-636. 26503923 10.1038/nrneurol.2015.197

[63] Schapira A, Chaudhuri K, Jenner P (2017). Non-motor features of Parkinson disease. Nature reviews. Neuroscience, 18:435-450. 28592904 10.1038/nrn.2017.62

[64] Liang C, Wang T, Luby-Phelps K, German D (2007). Mitochondria mass is low in mouse substantia nigra dopamine neurons: implications for Parkinson's disease. Experimental neurology, 203:370-380. 17010972 10.1016/j.expneurol.2006.08.015

[65] Fivenson EM, Lautrup S, Sun N, Scheibye-Knudsen M, Stevnsner T, Nilsen H, et al. (2017). Mitophagy in neurodegeneration and aging. Neurochem Int, 109:202-209. 28235551 10.1016/j.neuint.2017.02.007 PMC5565781

[66] Gredilla R, Bohr V, Stevnsner T (2010). Mitochondrial DNA repair and association with aging--an update. Experimental gerontology, 45:478-488. 20096766 10.1016/j.exger.2010.01.017 PMC2879476

[67] Rappold P, Tieu K (2010). Astrocytes and therapeutics for Parkinson's disease. Neurotherapeutics : the journal of the American Society for Experimental NeuroTherapeutics, 7:413-423. 20880505 10.1016/j.nurt.2010.07.001 PMC2948546

[68] Rodriguez M, Rodriguez-Sabate C, Morales I, Sanchez A, Sabate M (2015). Parkinson's disease as a result of aging. Aging cell, 14:293-308. 25677794 10.1111/acel.12312 PMC4406659

[69] Collins L, Toulouse A, Connor T, Nolan Y (2012). Contributions of central and systemic inflammation to the pathophysiology of Parkinson's disease. Neuropharmacology, 62:2154-2168. 22361232 10.1016/j.neuropharm.2012.01.028

[70] Bartels A (2011). Blood-brain barrier P-glycoprotein function in neurodegenerative disease. Current pharmaceutical design, 17:2771-2777. 21831040 10.2174/138161211797440122

[71] Mattson MP, Magnus T (2006). Ageing and neuronal vulnerability. Nat Rev Neurosci, 7:278-294. 16552414 10.1038/nrn1886 PMC3710114

[72] Rutten BP, Schmitz C, Gerlach OH, Oyen HM, de Mesquita EB, Steinbusch HW, et al. (2007). The aging brain: accumulation of DNA damage or neuron loss? Neurobiol Aging, 28:91-98. 16338029 10.1016/j.neurobiolaging.2005.10.019

[73] Ham PB 3rd, Raju R (2017). Mitochondrial function in hypoxic ischemic injury and influence of aging. Prog Neurobiol, 157:92-116. 27321753 10.1016/j.pneurobio.2016.06.006 PMC5161736

[74] Davis M, Whitely T, Turnbull D, Mendelow A (1997). Selective impairments of mitochondrial respiratory chain activity during aging and ischemic brain damage. Acta neurochirurgica. Supplement, 70:56-58. 10.1007/978-3-7091-6837-0_17 9416277

[75] Tatarková Z, Kuka S, Račay P, Lehotský J, Dobrota D, Mištuna D, et al. (2011). Effects of aging on activities of mitochondrial electron transport chain complexes and oxidative damage in rat heart. Physiological research, 60:281-289. 21114360 10.33549/physiolres.932019

[76] Xu K, Puchowicz M, Sun X, LaManna J (2008). Mitochondrial dysfunction in aging rat brain following transient global ischemia. Advances in experimental medicine and biology, 614:379-386. 18290349 10.1007/978-0-387-74911-2_42 PMC3071507

[77] He H, Lam M, McCormick T, Distelhorst C (1997). Maintenance of calcium homeostasis in the endoplasmic reticulum by Bcl-2. The Journal of cell biology, 138:1219-1228. 9298978 10.1083/jcb.138.6.1219 PMC2132547

[78] Ghavami S, Shojaei S, Yeganeh B, Ande SR, Jangamreddy JR, Mehrpour M, et al. (2014). Autophagy and apoptosis dysfunction in neurodegenerative disorders. Prog Neurobiol, 112:24-49. 24211851 10.1016/j.pneurobio.2013.10.004

[79] Chadwick W, Mitchell N, Martin B, Maudsley S (2012). Therapeutic targeting of the endoplasmic reticulum in Alzheimer's disease. Current Alzheimer research, 9:110-119. 22329655 10.2174/156720512799015055 PMC4682200

[80] Chen G, Gong M, Yan M, Zhang X (2013). Sevoflurane induces endoplasmic reticulum stress mediated apoptosis in hippocampal neurons of aging rats. PLoS One, 8:e57870. 23469093 10.1371/journal.pone.0057870 PMC3585271

[81] Salganik M, Sergeyev VG, Shinde V, Meyers CA, Gorbatyuk MS, Lin JH, et al. (2015). The loss of glucose-regulated protein 78 (GRP78) during normal aging or from siRNA knockdown augments human alpha-synuclein (alpha-syn) toxicity to rat nigral neurons. Neurobiol Aging, 36:2213-2223. 25863526 10.1016/j.neurobiolaging.2015.02.018 PMC4433578

[82] Blau C, Cowley T, O'Sullivan J, Grehan B, Browne T, Kelly L, et al. (2012). The age-related deficit in LTP is associated with changes in perfusion and blood-brain barrier permeability. Neurobiology of aging, 33:1005.e1023-1035. 10.1016/j.neurobiolaging.2011.09.035 22071124

[83] Stranahan AM, Jiam NT, Spiegel AM, Gallagher M (2012). Aging reduces total neuron number in the dorsal component of the rodent prefrontal cortex. J Comp Neurol, 520:1318-1326. 22020730 10.1002/cne.22790 PMC3931233

[84] Irwin RW, Brinton RD (2014). Allopregnanolone as regenerative therapeutic for Alzheimer's disease: translational development and clinical promise. Prog Neurobiol, 113:40-55. 24044981 10.1016/j.pneurobio.2013.08.004 PMC10124616

[85] Gemma C, Bachstetter A, Bickford P (2010). Neuron-Microglia Dialogue and Hippocampal Neurogenesis in the Aged Brain. Aging and disease, 1:232-244. 21961084 PMC3180926

[86] Smith LK, He Y, Park JS, Bieri G, Snethlage CE, Lin K, et al. (2015). beta2-microglobulin is a systemic pro-aging factor that impairs cognitive function and neurogenesis. Nat Med, 21:932-937. 26147761 10.1038/nm.3898 PMC4529371

[87] Rao MS, Hattiangady B, Shetty AK (2006). The window and mechanisms of major age-related decline in the production of new neurons within the dentate gyrus of the hippocampus. Aging Cell, 5:545-558. 17129216 10.1111/j.1474-9726.2006.00243.x

[88] Chaker Z, Aid S, Berry H, Holzenberger M (2015). Suppression of IGF-I signals in neural stem cells enhances neurogenesis and olfactory function during aging. Aging Cell, 14:847-856. 26219530 10.1111/acel.12365 PMC4568972

[89] Caspary DM, Ling L, Turner JG, Hughes LF (2008). Inhibitory neurotransmission, plasticity and aging in the mammalian central auditory system. J Exp Biol, 211:1781-1791. 18490394 10.1242/jeb.013581 PMC2409121

[90] Harris JL, Choi IY, Brooks WM (2015). Probing astrocyte metabolism in vivo: proton magnetic resonance spectroscopy in the injured and aging brain. Front Aging Neurosci, 7:202. 26578948 10.3389/fnagi.2015.00202 PMC4623195

[91] Rodriguez-Arellano JJ, Parpura V, Zorec R, Verkhratsky A (2016). Astrocytes in physiological aging and Alzheimer's disease. Neuroscience, 323:170-182. 25595973 10.1016/j.neuroscience.2015.01.007

[92] Vartak-Sharma N, Nooka S, Ghorpade A (2017). Astrocyte elevated gene-1 (AEG-1) and the A(E)Ging HIV/AIDS-HAND. Prog Neurobiol, 157:133-157. 27090750 10.1016/j.pneurobio.2016.03.006 PMC5982115

[93] Chisholm N, Sohrabji F (2016). Astrocytic response to cerebral ischemia is influenced by sex differences and impaired by aging. Neurobiology of disease, 85:245-253. 25843666 10.1016/j.nbd.2015.03.028 PMC5636213

[94] Diniz DG, Foro CA, Rego CM, Gloria DA, de Oliveira FR, Paes JM, et al. (2010). Environmental impoverishment and aging alter object recognition, spatial learning, and dentate gyrus astrocytes. Eur J Neurosci, 32:509-519. 20704596 10.1111/j.1460-9568.2010.07296.x

[95] Middeldorp J, Hol EM (2011). GFAP in health and disease. Prog Neurobiol, 93:421-443. 21219963 10.1016/j.pneurobio.2011.01.005

[96] David J, Ghozali F, Fallet-Bianco C, Wattez A, Delaine S, Boniface B, et al. (1997). Glial reaction in the hippocampal formation is highly correlated with aging in human brain. Neuroscience letters, 235:53-56. 9389594 10.1016/s0304-3940(97)00708-8

[97] Hwang ES, Yoon G, Kang HT (2009). A comparative analysis of the cell biology of senescence and aging. Cell Mol Life Sci, 66:2503-2524. 19421842 10.1007/s00018-009-0034-2 PMC11115533

[98] E G, Gonzalez J, Capani F, Morales L.2011. Role of Astrocytes in Neurodegenerative Diseases. In Neurodegenerative Diseases - Processes, Prevention, Protection and Monitoring.

[99] Bellaver B, Souza DG, Souza DO, Quincozes-Santos A (2017). Hippocampal Astrocyte Cultures from Adult and Aged Rats Reproduce Changes in Glial Functionality Observed in the Aging Brain. Mol Neurobiol, 54:2969-2985. 27026184 10.1007/s12035-016-9880-8

[100] Shi X, Wang B, Liu Y, Zhang J, Huang Y, Cao P, et al. (2017). Carnosine modulates glutamine synthetase expression in senescent astrocytes exposed to oxygen-glucose deprivation/recovery. Brain Res Bull, 130:138-145. 28115195 10.1016/j.brainresbull.2017.01.014

[101] Moftakhar P, Lynch M, Pomakian J, Vinters H (2010). Aquaporin expression in the brains of patients with or without cerebral amyloid angiopathy. Journal of neuropathology and experimental neurology, 69:1201-1209. 21107133 10.1097/NEN.0b013e3181fd252c PMC3155418

[102] Ishii T, Takanashi Y, Sugita K, Miyazawa M, Yanagihara R, Yasuda K, et al. (2017). Endogenous reactive oxygen species cause astrocyte defects and neuronal dysfunctions in the hippocampus: a new model for aging brain. Aging cell, 16:39-51. 27623715 10.1111/acel.12523 PMC5242301

[103] Zamanian JL, Xu L, Foo LC, Nouri N, Zhou L, Giffard RG, et al. (2012). Genomic analysis of reactive astrogliosis. J Neurosci, 32:6391-6410. 22553043 10.1523/JNEUROSCI.6221-11.2012 PMC3480225

[104] Simpson JE, Ince PG, Haynes LJ, Theaker R, Gelsthorpe C, Baxter L, et al. (2010). Population variation in oxidative stress and astrocyte DNA damage in relation to Alzheimer-type pathology in the ageing brain. Neuropathol Appl Neurobiol, 36:25-40. 19422529 10.1111/j.1365-2990.2009.01030.x

[105] Lee S, Wu Y, Shi XQ, Zhang J (2015). Characteristics of spinal microglia in aged and obese mice: potential contributions to impaired sensory behavior. Immun Ageing, 12:22. 26604973 10.1186/s12979-015-0049-5 PMC4657254

[106] Streit WJ, Xue QS (2014). Human CNS immune senescence and neurodegeneration. Curr Opin Immunol, 29:93-96. 24908174 10.1016/j.coi.2014.05.005

[107] Ojo JO, Rezaie P, Gabbott PL, Stewart MG (2015). Impact of age-related neuroglial cell responses on hippocampal deterioration. Front Aging Neurosci, 7:57. 25972808 10.3389/fnagi.2015.00057 PMC4413780

[108] Jimenez S, Baglietto-Vargas D, Caballero C, Moreno-Gonzalez I, Torres M, Sanchez-Varo R, et al. (2008). Inflammatory response in the hippocampus of PS1M146L/APP751SL mouse model of Alzheimer's disease: age-dependent switch in the microglial phenotype from alternative to classic. The Journal of neuroscience : the official journal of the Society for Neuroscience, 28:11650-11661. 18987201 10.1523/JNEUROSCI.3024-08.2008 PMC6671312

[109] Vaughan D, Peters A (1974). Neuroglial cells in the cerebral cortex of rats from young adulthood to old age: an electron microscope study. Journal of neurocytology, 3:405-429. 4373545 10.1007/BF01098730

[110] Hu X, Liou AK, Leak RK, Xu M, An C, Suenaga J, et al. (2014). Neurobiology of microglial action in CNS injuries: receptor-mediated signaling mechanisms and functional roles. Prog Neurobiol, 119-120:60-84. 24923657 10.1016/j.pneurobio.2014.06.002 PMC4121732

[111] Barakat R, Redzic Z (2016). The Role of Activated Microglia and Resident Macrophages in the Neurovascular Unit during Cerebral Ischemia: Is the Jury Still Out? Med Princ Pract, 25 Suppl 1:3-14. 10.1159/000435858 PMC5588523 26303836

[112] Palmer AL, Ousman SS (2018). Astrocytes and Aging. Front Aging Neurosci, 10:337. 30416441 10.3389/fnagi.2018.00337 PMC6212515

[113] Grabert K, Michoel T, Karavolos MH, Clohisey S, Baillie JK, Stevens MP, et al. (2016). Microglial brain region-dependent diversity and selective regional sensitivities to aging. Nat Neurosci, 19:504-516. 26780511 10.1038/nn.4222 PMC4768346

[114] Hickman SE, Kingery ND, Ohsumi TK, Borowsky ML, Wang LC, Means TK, et al. (2013). The microglial sensome revealed by direct RNA sequencing. Nat Neurosci, 16:1896-1905. 24162652 10.1038/nn.3554 PMC3840123

[115] Peters A (2009). The effects of normal aging on myelinated nerve fibers in monkey central nervous system. Front Neuroanat, 3:11. 19636385 10.3389/neuro.05.011.2009 PMC2713738

[116] Peters A.2007. The Effects of Normal Aging on Nerve Fibers and Neuroglia in the Central Nervous System. In Brain Aging: Models, Methods, and Mechanisms. Riddle DR, editor. Boca Raton (FL). 21204349

[117] Zhu X, Hill RA, Dietrich D, Komitova M, Suzuki R, Nishiyama A (2011). Age-dependent fate and lineage restriction of single NG2 cells. Development, 138:745-753. 21266410 10.1242/dev.047951 PMC3026417

[118] Sim F, Zhao C, Penderis J, Franklin R (2002). The age-related decrease in CNS remyelination efficiency is attributable to an impairment of both oligodendrocyte progenitor recruitment and differentiation. The Journal of neuroscience : the official journal of the Society for Neuroscience, 22:2451-2459. 11923409 10.1523/JNEUROSCI.22-07-02451.2002 PMC6758320

[119] Doucette JR, Jiao R, Nazarali AJ (2010). Age-related and cuprizone-induced changes in myelin and transcription factor gene expression and in oligodendrocyte cell densities in the rostral corpus callosum of mice. Cell Mol Neurobiol, 30:607-629. 20063055 10.1007/s10571-009-9486-z PMC11881803

[120] Linker RA, Lee DH, Demir S, Wiese S, Kruse N, Siglienti I, et al. (2010). Functional role of brain-derived neurotrophic factor in neuroprotective autoimmunity: therapeutic implications in a model of multiple sclerosis. Brain, 133:2248-2263. 20826430 10.1093/brain/awq179

[121] Lee Y, Morrison BM, Li Y, Lengacher S, Farah MH, Hoffman PN, et al. (2012). Oligodendroglia metabolically support axons and contribute to neurodegeneration. Nature, 487:443-448. 22801498 10.1038/nature11314 PMC3408792

[122] Donato AJ, Morgan RG, Walker AE, Lesniewski LA (2015). Cellular and molecular biology of aging endothelial cells. J Mol Cell Cardiol, 89:122-135. 25655936 10.1016/j.yjmcc.2015.01.021 PMC4522407

[123] Burns E, Kruckeberg T, Gaetano P (1981). Changes with age in cerebral capillary morphology. Neurobiology of aging, 2:283-291. 7335147 10.1016/0197-4580(81)90037-3

[124] Grammas P, Martinez J, Miller B (2011). Cerebral microvascular endothelium and the pathogenesis of neurodegenerative diseases. Expert Rev Mol Med, 13:e19. 21676288 10.1017/S1462399411001918

[125] Katusic ZS, Austin SA (2014). Endothelial nitric oxide: protector of a healthy mind. Eur Heart J, 35:888-894. 24357508 10.1093/eurheartj/eht544 PMC3977136

[126] Katusic ZS, Austin SA (2016). Neurovascular Protective Function of Endothelial Nitric Oxide- Recent Advances. Circ J, 80:1499-1503. 27238834 10.1253/circj.CJ-16-0423

[127] Raju K, Doulias PT, Evans P, Krizman EN, Jackson JG, Horyn O, et al. (2015). Regulation of brain glutamate metabolism by nitric oxide and S-nitrosylation. Sci Signal, 8:ra68. 26152695 10.1126/scisignal.aaa4312 PMC4746709

[128] Skoog I, Gustafson D (2006). Update on hypertension and Alzheimer's disease. Neurological research, 28:605-611. 16945211 10.1179/016164106X130506

[129] Solfrizzi V, Panza F, Colacicco A, D'Introno A, Capurso C, Torres F, et al. (2004). Vascular risk factors, incidence of MCI, and rates of progression to dementia. Neurology, 63:1882-1891. 15557506 10.1212/01.wnl.0000144281.38555.e3

[130] Springo Z, Toth P, Tarantini S, Ashpole NM, Tucsek Z, Sonntag WE, et al. (2015). Aging impairs myogenic adaptation to pulsatile pressure in mouse cerebral arteries. J Cereb Blood Flow Metab, 35:527-530. 25605292 10.1038/jcbfm.2014.256 PMC4420893

[131] Toth P, Tucsek Z, Tarantini S, Sosnowska D, Gautam T, Mitschelen M, et al. (2014). IGF-1 deficiency impairs cerebral myogenic autoregulation in hypertensive mice. J Cereb Blood Flow Metab, 34:1887-1897. 25248835 10.1038/jcbfm.2014.156 PMC4269740

[132] Blackwell KA, Sorenson JP, Richardson DM, Smith LA, Suda O, Nath K, et al. (2004). Mechanisms of aging-induced impairment of endothelium-dependent relaxation: role of tetrahydrobiopterin. Am J Physiol Heart Circ Physiol, 287:H2448-2453. 15319209 10.1152/ajpheart.00248.2004

[133] Prisby R, Ramsey M, Behnke B, Dominguez J, Donato A, Allen M, et al. (2007). Aging reduces skeletal blood flow, endothelium-dependent vasodilation, and NO bioavailability in rats. Journal of bone and mineral research : the official journal of the American Society for Bone and Mineral Research, 22:1280-1288. 10.1359/jbmr.070415 17451371

[134] Chauhan A, More R, Mullins P, Taylor G, Petch C, Schofield P (1996). Aging-associated endothelial dysfunction in humans is reversed by L-arginine. Journal of the American College of Cardiology, 28:1796-1804. 8962569 10.1016/s0735-1097(96)00394-4

[135] Schulze F, Maas R, Freese R, Schwedhelm E, Silberhorn E, Böger R (2005). Determination of a reference value for N(G), N(G)-dimethyl-L-arginine in 500 subjects. European journal of clinical investigation, 35:622-626. 16178881 10.1111/j.1365-2362.2005.01561.x

[136] Puca A, Carrizzo A, Ferrario A, Villa F, Vecchione C (2012). Endothelial nitric oxide synthase, vascular integrity and human exceptional longevity. Immunity & ageing : I & A, 9:26. 23153280 10.1186/1742-4933-9-26 PMC3538508

[137] Gradinaru D, Borsa C, Ionescu C, Prada GI (2015). Oxidized LDL and NO synthesis--Biomarkers of endothelial dysfunction and ageing. Mech Ageing Dev, 151:101-113. 25804383 10.1016/j.mad.2015.03.003

[138] Koike T, Vernon R, Gooden M, Sadoun E, Reed M (2003). Inhibited angiogenesis in aging: a role for TIMP-2. The journals of gerontology. Series A, Biological sciences and medical sciences, 58:B798-805. 14528035 10.1093/gerona/58.9.b798

[139] Lahteenvuo J, Rosenzweig A (2012). Effects of aging on angiogenesis. Circ Res, 110:1252-1264. 22539758 10.1161/CIRCRESAHA.111.246116 PMC4101916

[140] Hicks P, Rolsten C, Brizzee D, Samorajski T (1983). Age-related changes in rat brain capillaries. Neurobiology of aging, 4:69-75. 6877490 10.1016/0197-4580(83)90057-x

[141] Sturrock R (1980). A comparative quantitative and morphological study of ageing in the mouse neostriatum, indusium griseum and anterior commissure. Neuropathology and applied neurobiology, 6:51-68. 7374912 10.1111/j.1365-2990.1980.tb00204.x

[142] Peters A, Josephson K, Vincent S (1991). Effects of aging on the neuroglial cells and pericytes within area 17 of the rhesus monkey cerebral cortex. The Anatomical record, 229:384-398. 2024779 10.1002/ar.1092290311

[143] Knox C, Yates R, Chen I, Klara P (1980). Effects of aging on the structural and permeability characteristics of cerebrovasculature in normotensive and hypertensive strains of rats. Acta neuropathologica, 51:1-13. 7435136 10.1007/BF00688844

[144] Tigges J, Herndon J, Rosene D (1995). Mild age-related changes in the dentate gyrus of adult rhesus monkeys. Acta anatomica, 153:39-48. 8560958 10.1159/000147713

[145] Peinado M, Quesada A, Pedrosa J, Torres M, Martinez M, Esteban F, et al. (1998). Quantitative and ultrastructural changes in glia and pericytes in the parietal cortex of the aging rat. Microscopy research and technique, 43:34-42. 9829457 10.1002/(SICI)1097-0029(19981001)43:1<34::AID-JEMT6>3.0.CO;2-G

[146] de Jong G, Horváth E, Luiten P (1990). Effects of early onset of nimodipine treatment on microvascular integrity in the aging rat brain. Stroke, 21:IV113-116. 2260133

[147] Stewart P, Magliocco M, Hayakawa K, Farrell C, Del Maestro R, Girvin J, et al. (1987). A quantitative analysis of blood-brain barrier ultrastructure in the aging human. Microvascular research, 33:270-282. 3587079 10.1016/0026-2862(87)90022-7

[148] Bell RD, Winkler EA, Sagare AP, Singh I, LaRue B, Deane R, et al. (2010). Pericytes control key neurovascular functions and neuronal phenotype in the adult brain and during brain aging. Neuron, 68:409-427. 21040844 10.1016/j.neuron.2010.09.043 PMC3056408

[149] Heinsen H, Heinsen Y (1983). Cerebellar capillaries. Qualitative and quantitative observations in young and senile rats. Anatomy and embryology, 168:101-116. 6650851 10.1007/BF00305402

[150] Yamazaki Y, Baker DJ, Tachibana M, Liu CC, van Deursen JM, Brott TG, et al. (2016). Vascular Cell Senescence Contributes to Blood-Brain Barrier Breakdown. Stroke, 47:1068-1077. 26883501 10.1161/STROKEAHA.115.010835 PMC4811685

[151] Belanger M, Allaman I, Magistretti PJ (2011). Brain energy metabolism: focus on astrocyte-neuron metabolic cooperation. Cell Metab, 14:724-738. 22152301 10.1016/j.cmet.2011.08.016

[152] Brooks GA (2009). Cell-cell and intracellular lactate shuttles. J Physiol, 587:5591-5600. 19805739 10.1113/jphysiol.2009.178350 PMC2805372

[153] Pellerin L, Magistretti P (1994). Glutamate uptake into astrocytes stimulates aerobic glycolysis: a mechanism coupling neuronal activity to glucose utilization. Proceedings of the National Academy of Sciences of the United States of America, 91:10625-10629. 7938003 10.1073/pnas.91.22.10625 PMC45074

[154] Abe T, Takahashi S, Suzuki N (2006). Oxidative metabolism in cultured rat astroglia: effects of reducing the glucose concentration in the culture medium and of D-aspartate or potassium stimulation. J Cereb Blood Flow Metab, 26:153-160. 15973351 10.1038/sj.jcbfm.9600175

[155] Mergenthaler P, Lindauer U, Dienel GA, Meisel A (2013). Sugar for the brain: the role of glucose in physiological and pathological brain function. Trends Neurosci, 36:587-597. 23968694 10.1016/j.tins.2013.07.001 PMC3900881

[156] Zhao Y, Fung C, Shin D, Shin BC, Thamotharan S, Sankar R, et al. (2010). Neuronal glucose transporter isoform 3 deficient mice demonstrate features of autism spectrum disorders. Mol Psychiatry, 15:286-299. 19506559 10.1038/mp.2009.51 PMC4208914

[157] Souza DG, Bellaver B, Raupp GS, Souza DO, Quincozes-Santos A (2015). Astrocytes from adult Wistar rats aged in vitro show changes in glial functions. Neurochem Int, 90:93-97. 26210720 10.1016/j.neuint.2015.07.016

[158] Ding F, Yao J, Rettberg JR, Chen S, Brinton RD (2013). Early decline in glucose transport and metabolism precedes shift to ketogenic system in female aging and Alzheimer's mouse brain: implication for bioenergetic intervention. PLoS One, 8:e79977. 24244584 10.1371/journal.pone.0079977 PMC3823655

[159] Fattoretti P, Bertoni-Freddari C, Casoli T, Di Stefano G, Solazzi M, Giorgetti B (2002). Decreased expression of glucose transport protein (Glut3) in aging and vitamin E deficiency. Annals of the New York Academy of Sciences, 973:293-296. 12485881 10.1111/j.1749-6632.2002.tb04653.x

[160] Boumezbeur F, Mason GF, de Graaf RA, Behar KL, Cline GW, Shulman GI, et al. (2010). Altered brain mitochondrial metabolism in healthy aging as assessed by in vivo magnetic resonance spectroscopy. J Cereb Blood Flow Metab, 30:211-221. 19794401 10.1038/jcbfm.2009.197 PMC2949111

[161] Jiang T, Cadenas E (2014). Astrocytic metabolic and inflammatory changes as a function of age. Aging Cell, 13:1059-1067. 25233945 10.1111/acel.12268 PMC4244278

[162] Rosko L, Smith VN, Yamazaki R, Huang JK (2019). Oligodendrocyte Bioenergetics in Health and Disease. Neuroscientist, 25:334-343. 30122106 10.1177/1073858418793077 PMC6745601

[163] Funfschilling U, Supplie LM, Mahad D, Boretius S, Saab AS, Edgar J, et al. (2012). Glycolytic oligodendrocytes maintain myelin and long-term axonal integrity. Nature, 485:517-521. 22622581 10.1038/nature11007 PMC3613737

[164] Zeis T, Allaman I, Gentner M, Schroder K, Tschopp J, Magistretti PJ, et al. (2015). Metabolic gene expression changes in astrocytes in Multiple Sclerosis cerebral cortex are indicative of immune-mediated signaling. Brain Behav Immun, 48:313-325. 25937052 10.1016/j.bbi.2015.04.013

[165] Cambron M, D'Haeseleer M, Laureys G, Clinckers R, Debruyne J, De Keyser J (2012). White-Matter Astrocytes, Axonal Energy Metabolism, and Axonal Degeneration in Multiple Sclerosis. Journal of Cerebral Blood Flow & Metabolism, 32:413-424. 22214904 10.1038/jcbfm.2011.193 PMC3293127

[166] Saab AS, Nave KA (2017). Myelin dynamics: protecting and shaping neuronal functions. Curr Opin Neurobiol, 47:104-112. 29065345 10.1016/j.conb.2017.09.013

[167] Liu Y, Li M, Zhang Z, Ye Y, Zhou J (2018). Role of microglia-neuron interactions in diabetic encephalopathy. Ageing Res Rev, 42:28-39. 29247713 10.1016/j.arr.2017.12.005

[168] Kalsbeek MJ, Mulder L, Yi CX (2016). Microglia energy metabolism in metabolic disorder. Mol Cell Endocrinol, 438:27-35. 27687525 10.1016/j.mce.2016.09.028

[169] Payne J, Maher F, Simpson I, Mattice L, Davies P (1997). Glucose transporter Glut 5 expression in microglial cells. Glia, 21:327-331. 9383041 10.1002/(sici)1098-1136(199711)21:3<327::aid-glia7>3.0.co;2-1

[170] Samorajski T (1976). How the human brain responds to aging. Journal of the American Geriatrics Society, 24:4-11. 172540 10.1111/j.1532-5415.1976.tb03246.x

[171] Schuitemaker A, van der Doef TF, Boellaard R, van der Flier WM, Yaqub M, Windhorst AD, et al. (2012). Microglial activation in healthy aging. Neurobiol Aging, 33:1067-1072. 21051106 10.1016/j.neurobiolaging.2010.09.016

[172] Perry V, Matyszak M, Fearn S (1993). Altered antigen expression of microglia in the aged rodent CNS. Glia, 7:60-67. 8423063 10.1002/glia.440070111

[173] Valles SL, Iradi A, Aldasoro M, Vila JM, Aldasoro C, de la Torre J, et al. (2019). Function of Glia in Aging and the Brain Diseases. Int J Med Sci, 16:1473-1479. 31673239 10.7150/ijms.37769 PMC6818212

[174] Letiembre M, Hao W, Liu Y, Walter S, Mihaljevic I, Rivest S, et al. (2007). Innate immune receptor expression in normal brain aging. Neuroscience, 146:248-254. 17293054 10.1016/j.neuroscience.2007.01.004

[175] Frank MG, Barrientos RM, Biedenkapp JC, Rudy JW, Watkins LR, Maier SF (2006). mRNA up-regulation of MHC II and pivotal pro-inflammatory genes in normal brain aging. Neurobiol Aging, 27:717-722. 15890435 10.1016/j.neurobiolaging.2005.03.013

[176] Craft S (2006). Insulin resistance syndrome and Alzheimer disease: pathophysiologic mechanisms and therapeutic implications. Alzheimer disease and associated disorders, 20:298-301. 17132977 10.1097/01.wad.0000213866.86934.7e

[177] Eyo UB, Wu LJ (2013). Bidirectional microglia-neuron communication in the healthy brain. Neural Plast, 2013:456857. 24078884 10.1155/2013/456857 PMC3775394

[178] Shih AY, Fernandes HB, Choi FY, Kozoriz MG, Liu Y, Li P, et al. (2006). Policing the police: astrocytes modulate microglial activation. J Neurosci, 26:3887-3888. 16611803 10.1523/JNEUROSCI.0936-06.2006 PMC6673885

[179] Flowers A, Bell-Temin H, Jalloh A, Stevens SM Jr, Bickford PC (2017). Proteomic anaysis of aged microglia: shifts in transcription, bioenergetics, and nutrient response. J Neuroinflammation, 14:96. 28468668 10.1186/s12974-017-0840-7 PMC5415769

[180] Lundgaard I, Li B, Xie L, Kang H, Sanggaard S, Haswell JD, et al. (2015). Direct neuronal glucose uptake heralds activity-dependent increases in cerebral metabolism. Nat Commun, 6:6807. 25904018 10.1038/ncomms7807 PMC4410436

[181] Itoh Y, Abe T, Takaoka R, Tanahashi N (2004). Fluorometric determination of glucose utilization in neurons in vitro and in vivo. J Cereb Blood Flow Metab, 24:993-1003. 15356420 10.1097/01.WCB.0000127661.07591.DE

[182] Hladky SB, Barrand MA (2016). Fluid and ion transfer across the blood-brain and blood-cerebrospinal fluid barriers; a comparative account of mechanisms and roles. Fluids Barriers CNS, 13:19. 27799072 10.1186/s12987-016-0040-3 PMC5508927

[183] Mooradian A, Morin A, Cipp L, Haspel H (1991). Glucose transport is reduced in the blood-brain barrier of aged rats. Brain research, 551:145-149. 1913147 10.1016/0006-8993(91)90926-m

[184] Gschanes A, Boado R, Sametz W, Windisch M (2000). The drug cerebrolysin and its peptide fraction E021 increase the abundance of the blood-brain barrier GLUT1 glucose transporter in brains of young and old rats. The Histochemical journal, 32:71-77. 10816070 10.1023/a:1004003008683

[185] Vorbrodt A, Dobrogowska D, Meeker H, Carp R (1999). Immunogold study of regional differences in the distribution of glucose transporter (GLUT-1) in mouse brain associated with physiological and accelerated aging and scrapie infection. Journal of neurocytology, 28:711-719. 10859574 10.1023/a:1007034003114

[186] Perez-Escuredo J, Van Hee VF, Sboarina M, Falces J, Payen VL, Pellerin L, et al. (2016). Monocarboxylate transporters in the brain and in cancer. Biochim Biophys Acta, 1863:2481-2497. 26993058 10.1016/j.bbamcr.2016.03.013 PMC4990061

